# Ultrasound as a First-Line Modality for Acute Colonic Diverticulitis: A Prospective Comparison with CT

**DOI:** 10.3390/jcm14072510

**Published:** 2025-04-07

**Authors:** Gil N. Bachar, Eli Atar, Moran Dahan, Haim Neiman, Tamar Gurvitz, Issa Nidal, Selma Gabrieli

**Affiliations:** 1 Department of Diagnostic Imaging, Ultrasound and Computed Tomography Units, Petah Tiqva 49100, Israel; elia@clalit.org.il (E.A.); yomoranda@clalit.org.il (M.D.); haimn@clalit.org.il (H.N.); tamarpa12@clalit.org.il (T.G.); selmag@clalit.org.il (S.G.); 2 Department of Surgery B, Rabin Medical Center-Hashron Hospital, Petach Tikva, and Sackler Faculty of Medicine, Tel Aviv University, Tel Aviv, Israel; nidalissa@clalit.org.il

**Keywords:** diverticulitis, ultrasonography, computed tomography, sensitivity and specificity, diagnosis

## Abstract

**Objectives:** We aimed to compare the accuracy of ultrasound and computed tomography (CT) for the diagnosis of patients with suspected acute diverticulitis and to determine if ultrasound might serve as the primary tool for this purpose in the emergency department. **Methods:** A double-blind prospective study design was used. The study group included 142 consecutive patients with clinically suspected diverticulitis admitted to the emergency department of a tertiary medical center in 2016–2019. All underwent first ultrasound examination followed by abdominal CT. The final diagnosis was interpreted independently by an expert radiologist in a blinded fashion. Imaging data were compared with final diagnosis and we analyzed the findings against the medical, clinical, and laboratory data. **Results:** The final diagnosis was colonic diverticulitis in 98 patients. Sensitivity was 93.8% for ultrasound and 100% for CT; corresponding specificity rates were 86.7% and 100%. Agreement between the modalities was excellent (kappa = 0.81). CT demonstrated complicated diverticulosis in 18 patients: 8 pericolic abscesses, 9 micro-perforations, and 1 fistula. Ultrasound missed one abscess and five micro-perforations; however, all were small and were treated conservatively. Twenty-three patients were found to have an acute abdominal condition other than diverticulitis; sensitivity in these cases was 60.8% for ultrasound and 91.3% for CT. In 21 patients, the diagnosis was unknown. **Conclusions:** Ultrasound has similar sensitivity and specificity to CT for the diagnosis of acute colonic diverticulitis. We believe ultrasound may serve as the initial imaging modality in the emergency department, with CT reserved for large abscesses or inconclusive ultrasound findings.

## 1. Introduction

Colonic diverticula are formed by acquired herniation of the mucosa through a defect in the colonic wall. In 10–25% of patients, the orifice of a diverticulum becomes obstructed and inflamed, leading to diverticulitis. Diverticular disease is common in the Western world. It affects people after the fourth decade, with a prevalence of 70% in the over-65-year age group and up to 80% in the over-80-year age group [1,2].

Identifying patients with acute diverticulitis in the emergency department can be challenging. Previous studies have shown that diagnoses based on clinical parameters alone are often erroneous and may lead to delayed or unnecessary treatment and, consequently, extra morbidity or mortality [2,3]. Currently, the American College of Radiology [4] and the American Society of Colon and Rectum Surgeons [5] recommend abdominal and pelvis computed tomography (CT) with contrast medium as the initial examination for patients with suspected acute diverticulitis. However, others suggested that ultrasound should be the primary imaging technique because of its wide availability, low cost, and high diagnostic sensitivity (84–100%) [6,7,8,9,10,11]. Moreover, it spares patients the ionizing radiation of CT and eliminates the need for contrast medium, especially in patients with iodine allergy or renal failure.

To our knowledge, there are only a small number of studies directly comparing the value of ultrasound and CT for the diagnosis of acute diverticulitis. The aim of the present prospective study was to investigate the diagnostic accuracy and advantages and limitations of ultrasound relative to CT in patients referred to the emergency department for evaluation of suspected acute diverticulitis. In addition, we sought to determine the ability of ultrasound to differentiate between simple and complicated diverticulitis.

## 2. Materials and Methods

### 2.1. Patients

A prospective study design was used. The study group consisted of consecutive patients who presented to the emergency department of a tertiary university-affiliated medical center with clinically suspected acute diverticulitis between August 2016 and October 2019 and were referred to the radiology department for further evaluation. Exclusion criteria were age under 18 years and current pregnancy. Eligible patients were invited to participate in the study after being orally informed that an additional ultrasound examination would be performed prior to the diagnostic CT. Consenting patients were included in the study. The study was conducted in accordance with the Helsinki Declaration and approved by the local Institutional Review Board of Rabin Medical Center (RMC-0503-16-RMC).

### 2.2. Procedure

At presentation, patients underwent clinical assessment in the emergency department followed within a few hours by ultrasound and CT in the radiology department. To guarantee a blinded evaluation, ultrasound was performed first by an experienced board-certified sonographer (G.S., B.N.G., N.H.) and then CT was performed, in a separate unit, by a board-certified abdominal radiologist (B.N.G., A.E) who was blinded to the ultrasound findings. In no case was the ultrasound examination performed by a technologist.

### 2.3. Ultrasound

Ultrasound was performed with the Acuson S2000 system (Siemens, Erlangen, Germany) equipped with 3–6 MHz convex-array or 7–9 MHz linear-array transducers. Initially, the colon and adjacent zones were explored, particularly the area that the patient reported was painful. This was followed by scanning with a 6–10 MHz transducer using a graded compression technique with special attention to the zone that was causing pain [12]. We also used color Doppler and power Doppler to obtain additional information about the inflamed bowel wall, quantify intestinal wall vascularization, and visualize the hyperdynamic splanchnic blood flow that is characteristic of acute inflammation. At completion of these studies, a general examination of the abdominal cavity was conducted with a 3–6 MHz convex-array transducer, including a search for free fluid and signs of pneumoperitoneum. When no abnormalities were found, a detailed examination was undertaken to determine an alternative diagnosis.

### 2.4. Computed Tomography

CT procedures were performed using a 128-slice multidetector scanner (Definition Flash, Siemens) with the following specifications: tube voltage of 120 Kv, quality reference tube current–time product of 110 mAs, rotation time of 0.5 s, and pitch of 1.2. Scans were obtained under inspiratory breath-hold in the cranio-caudal direction. Collimation was 128 × 0.6 mm, and reconstruction slice width was 3 mm. The CT protocol included 1000 cc of water-soluble contrast material administered orally 2 h before scanning (Gastrografin; Bristol-Myers Squib, Wallingford, CT, USA), 100 mL of nonionic contrast agent (Ultravist 300; Schering, Berlin, Germany) injected into the antecubital vein at a rate of 3–4 mL/s using a dual-head automatic injector (Stellant; Medrad, Warrendale, PA, USA), and a 30 mL saline flush. Rectal contrast agent was not used.

### 2.5. Analysis

The evaluation of the colon wall and adjacent zone on the ultrasound and CT scans focused on five criteria: bowel wall thickening more than 3 mm, diverticula, inflammatory pericolonic fat, abscess, and extraluminal pericolonic air. Findings were considered positive if imaging yielded at least one mural sign (bowel wall thickening or diverticula) together with at least one extramural sign (inflammatory pericolic fat, hyperemia on Doppler scanning, abscess, or extraluminal air). Diverticulitis was classified as simple if there was thickening of the bowel wall, inflamed diverticula, hyperemia on Doppler scanning, or stranding of pericolic fat, and complicated if there was abscess formation, stenosis, fistula, free fluid, or extraluminal free air. Both CT and ultrasound procedures included an evaluation of all abdominal organs: liver, spleen, gallbladder, bile duct, pancreas, kidneys, gastrointestinal tract, appendix, lymph nodes, peritoneum, mesentery, bladder, prostate, pelvis, and female genitalia.

The final diagnosis was made by a panel consisting of an experienced abdominal radiologist and an experienced gastrointestinal surgeon who interpreted the scans individually in a blinded manner. In addition to imaging findings, the diagnosis was based on clinical parameters and response to medical treatment (n = 91), laboratory findings (n = 142), and findings on colonoscopy (n = 49), surgery (n = 8), histopathologic study (n = 56) and 6-month outpatient follow-up data. A consensus was reached if their diagnoses disagreed.

### 2.6. Statistical Analysis

The primary outcome measure was the accuracy of ultrasound compared to CT for the diagnosis of acute diverticulitis in the emergency department. The sensitivity, specificity, and positive and negative predictive values (PPV, NPV) were calculated for US and CT, and the agreement between the modalities was analyzed with kappa statistics. Strength of agreement was classified as slight (kappa ≤ 20), fair (kappa 0.21–0.40), moderate (kappa 0.61–0.8), and excellent (kappa 0.81–1.00). Differences in predictive values between the imaging modalities were analyzed with chi-squared test. The ability to establish an alternative diagnosis using ultrasound and CT was determined as well. Continuous variables were compared using Student’s *t* test, and categorical variables were compared by cross-table analysis using chi-squared or Fisher’s exact test, as appropriate. A confidence interval of 95% was chosen. Differences were considered statistically significant when *p* values were less than 0.05. All calculations were performed with SPSS version 21.0 (SPSS Inc., Chicago, IL, USA).

## 3. Results

The study group consisted of 142 patients with clinically suspected diverticulitis, 69 women and 73 men, aged 20–90 years (mean 60 ± 16.3 years).

The final diagnosis was acute diverticulitis in 98 patients (69%), localized to the sigmoid colon in 85, cecum in 2, ascending colon, hepatic flexure, or transverse colon in 1 each, and descending colon in 8. Table 1 shows the accuracy of CT and ultrasound for the diagnosis of acute diverticulitis. For CT, sensitivity was 100% (CI 95% 95.3–100), specificity was 100% (CI 95% 90–100), accuracy was 100%, PPV was 100% (CI 95% 95–100), and NPV was 100% (CI 95% 90–100), with 98 true positives and 44 true negatives. For ultrasound, sensitivity was 93.8% (CI 95% 86.6–97.5), specificity was 86.7% (CI 95% 72.2–89.6), accuracy was 89.4%, PPV 91% (CI 95% 83.3–95.6), and NPV was 85.3% (CI 95% 70.1–93.9), with 92 true positives, 6 false negatives, 9 false positives, and 35 true negatives. Although ultrasound failed to identify six cases of acute diverticulitis, agreement between the modalities was excellent, with a kappa value of 0.81 (CI 95% 0.70–0.91). All cases of acute colonic diverticulitis were managed conservatively.

In total, 80 patients (82%) had simple diverticulitis and 18 had complicated diverticulitis. Complications identified by CT included pericolic abscess in eight patients (8.1%) (mean diameter 3.9 cm, range 1.3–8.0 cm), micro-perforation in nine (9.1%), and fistula (between the sigma and bladder) in one. Ultrasound missed one abscess measuring 1.5 cm in diameter and five micro-perforations, all small. The sensitivity of ultrasound for detecting complicated diverticulitis was 61.1% (CI 95% 36.1–81.7), the specificity was 95% (CI 95% 87.0–98.3), accuracy was 88.7%, PPV was 73.3% (CI 95% 44.8–91.0), and NPV was 91.5% (CI 95% 82.8–96.2), with 11 true positives, 7 false negatives, 4 false positives, and 76 true negatives. Abscesses were treated conservatively with antibiotics in seven cases and by percutaneous CT-guided drainage in one. All micro-perforations were treated conservatively.

Hospital stay was significantly shorter for patients with simple diverticulitis (mean 4.31 ± 2.1 days, range 0–9 days) than patients with complicated diverticulitis (mean 7.05 ± 2.5 days, range 3–12 days) (*p* < 0.0001). All patients were followed at the outpatient clinic 2–4 weeks after discharge. Fifty-two patients (53%) underwent colonoscopy within 6 months after discharge; signs of diverticular disease were found in all cases.

Acute abdominal conditions other than diverticulitis were diagnosed in 23 patients, as listed in Table 2. The sensitivity of CT for the diagnosis of an alternative pathology was 91.3% (CI 95% 70.5–98.5), specificity was 95.2% (CI 95% 74.1–99.7), and accuracy was 93.2%, with 21 true positives, 2 false negatives, 1 false positive, and 20 true negatives. CT failed to identify one case of colitis and one case of gastroenteritis. The sensitivity of ultrasound for identifying alternative diagnoses was 60.8% (CI 95% 38.7–79.5), specificity was 80.9% (CI 95% 57.4–93.7), and accuracy was 93.2%, with 14 true positives, 9 false negatives, 4 false positives, and 17 true negatives.

In the remaining 21 patients, the diagnosis was abdominal pain of unknown cause.

The CT scanner recorded the effective radiation dose to which patients were exposed. The mean dose per patient in the 142 examinations was 5 ± 1.2 mSv.

## 4. Discussion

The clinical diagnosis and assessment of acute colonic diverticulitis can be difficult [2,13] because the classic pattern of left lower quadrant pain, tenderness, fever, and leukocytosis can be mimicked by numerous other acute abdominal conditions. The reported sensitivity of the clinical diagnosis of acute diverticulitis was only 68% of 802 patients in the prospective study of Toorenvliet et al. [2] and 64% in the study of Laurell et al. [14]. Hackford et al. [15] found that of 140 patients with acute diverticulitis, only 35 (25%) presented with fever and up to 90 (64%) had a white blood cell count within normal range. In the present study, the clinical diagnosis had a sensitivity of 69%; thus, 31% of the patients were misdiagnosed. Even higher rates of misdiagnosis (34–67%) have been reported by others [3,16].

Therefore, additional radiological evaluation is needed to confirm the diagnosis and to distinguish patients with acute diverticulitis from those with another acute abdominal condition that produces the same symptoms. In the Western world, CT is considered the diagnostic modality of choice because of its high sensitivity, specificity, reproducibility, and availability [17]. Furthermore, if abscesses are detected, CT may be used to guide percutaneous drainage and identify embolization of diverticular bleeding. The American College of Radiology recommends CT of the abdomen and pelvis as the preferred procedure in the setting of left lower quadrant pain with or without fever, except in women of childbearing age in whom ultrasound is the initial preferred modality [18]. Accordingly, in prospective studies of consecutive patients with suspected colonic diverticulitis, Pradel et al. [13] found that CT had a sensitivity of 91% and specificity of 77%, and Cho et al. [19] reported a similar sensitivity of 93% upon comparison of CT with barium enema. Overall, the reported sensitivity of CT for the diagnosis of diverticulitis ranges between 79% and 99% with specificity approaching 100% [11]. In the present study, the sensitivity and specificity of CT were 100%.

However, CT has several important disadvantages compared to ultrasound. It is less accessible in emerging and undeveloped countries, requires more time for interpretation of the findings by a radiologist, is more costly, and exposes patients to ionizing radiation and injection of oral, rectal, or intravenous contrast medium [20]. The last consideration is particularly important given that diverticulitis is prone to recurrence and patients may require several CT examinations, placing them at increased risk of cancer. Furthermore, ultrasound can be performed at the bedside in the emergency department. The spatial resolution of high-frequency ultrasound images is high, especially when a graded compression technique is used [10,11,13,21]. For these reasons, we hypothesized that CT might be replaced with ultrasound in the emergency department when feasible or if CT is not available.

The findings of the present study comparing ultrasound and CT support this practice. Ultrasound had a sensitivity of 93.8%, specificity of 86.7%, accuracy of 89.4%, PPV of 91%, and NPV of 85.3%, and the agreement with CT was excellent (kappa = 0.81). Similar results have been published in the literature. The meta-analysis of Laméris et al. [11] revealed no significant differences in rates of sensitivity, specificity and accuracy between ultrasound and CT, results that were sustained several years later in a study of 253 patients with suspected simple diverticulitis by King et al. [21]. Others reported ultrasound sensitivity rates ranging from 77% to 98% and specificity rates from 80% to 99% [2,9,10,13,22].

Complicated diverticulitis was found in 18 patients (18%) in our cohort. Ultrasound missed one of eight pericolic abscesses detected by CT and five of nine cases of micro-perforations. However, in no case was the missed diagnosis clinically significant in terms of patient care: both the missed abscess (1.5 cm in diameter) and the perforations were quite small and treatable with conservative measures and had no effect on patient care. One fistula between the sigmoid and bladder was missed by ultrasound as well. The sensitivity of ultrasound for the detection of complicated diverticulitis was 61.1%; the specificity was 95% and accuracy was 88.7%. Because ultrasound is operator-dependent, proper technique is crucial for an accurate diagnosis. Our findings suggest that CT is better for detecting small extraluminal air bubbles indicative of micro-perforations. However, ultrasound can provide equally useful data regarding management of diverticulitis complicated by abscess. CT should be reserved for patients with large abscesses requiring percutaneous drainage, suspected fistula, or an inconclusive ultrasound examination [20].

The initial ultrasound findings may be predictive of length of hospital stay, which was about 2.5 days shorter for the patients with simple diverticulitis than those with complicated diverticulitis (*p* < 0.0001).

Accurate detection of another pathology when colonic diverticulitis is not the correct diagnosis is an equally important goal of CT. In our study, CT was more useful than ultrasound in these cases, with a sensitivity of 91.3% compared to 60.8% for ultrasound, and a specificity of 95.2% compared to 80.9%. These rates are in line with earlier reports in the literature [13,22] and agree with the conclusion of the meta-analysis by Laméris et al. [11]. In our experience, CT allows for quick assessment of intraperitoneal and retroperitoneal organs and the abdominal wall and pelvis. Since the quality of ultrasound is largely dependent on the skills of the operator, its accuracy could be lower in specific subgroups such as obese patients, older and uncooperative patients, and pregnant women.

Findings in the medical literature for radiation-induced cancer rates vary widely. According to some studies, approximately 10% of all lifetime cancer cases are attributable to radiation [23]. In the present study, had CT not been used in uncomplicated cases, 82% of patients (80/142) would have been spared a mean effective radiation dose of 5 ± 1.2 mSv, or a total of 400 mSv for the whole study population.

This study has several potential limitations. The study was conducted in a single university-affiliated tertiary center, and all sonograms were performed by experienced board-certified sonographers. Therefore, it is possible that the diagnostic accuracy was higher than might be expected in small, lower-level hospitals. Nevertheless, Zielke et al. [24] found that even a few months’ training in abdominal ultrasound was sufficient to achieve equal diagnostic accuracy to that reported in studies using experienced observers. Another limitation of our study is the lack of pathologic correlation because the majority of patients had conservative treatment. Therefore, the final diagnosis was established by an expert panel who individually evaluated the clinical data, physical examination, laboratory findings, imaging scans, and response to medical treatment of each patient.

In conclusion, ultrasound has similar sensitivity and specificity to CT for the diagnosis of colonic diverticulitis. It is rapid, accurate, and widely available, and does not require ionizing radiation. Its low cost compared to CT can lead to considerable savings for healthcare services. In light of our results and other publications [13,19], we believe that ultrasound may serve as the initial imaging modality for patients referred to the emergency department with suspected acute colonic diverticulitis if CT is not available. Abdominal CT should be reserved for patients with generalized peritonitis, large abscesses requiring percutaneous drainage, fistula, or inconclusive ultrasound findings [13].

## Figures and Tables

**Table 1:** Accuracy of ultrasound and CT for the diagnosis of colonic diverticulitis.

	Sensitivity	Specificity	Accuracy	PPV	NPV
Ultrasound diverticulitis	93.8%	86.7%	89.4%	91%	85.3%
CT diverticulitis	100%	100%	100%	100%	100%
Ultrasound complicated diverticulitis	61.1%	95%	88.7%	73.3%	91.5%
Ultrasound alternative diagnosis	60.8%	80.9%	93.2%		

PPV, positive predictive value; NPV, negative predictive value; CT, computed tomography; US, ultrasound.

**Table 2:** Alternative diagnoses in 23 of 142 patients clinically suspected of having acute diverticulitis.

Alternative Diagnosis	No. of Patients
Colitis	3
Abscess of small bowel	1
Ureterolithiasis and hydronephrosis	4
Retroperitoneal mass	1
Small bowel obstruction	1
Mesenteric panniculitis	1
Carcinoma of sigma	1
Abdominal wall hematoma	1
Vasculitis	1
Epiploic appendagitis	5
Gastroenteritis	1
Periappendicular abscess	1
Appendicitis	1
Inguinal hernia	1

## Data Availability

Data is unavailable due to privacy or ethical restrictions.

## Abbreviations

CI	confidence interval
CT	computed tomography
NPV	negative predictive value
PPV	positive predictive value
